# Preliminary Assessment of a Hybrid Implant Design Submitted to Immediate Placement with Abutment Exposure: A Pilot Study in One Dog Model

**DOI:** 10.3390/dj13100463

**Published:** 2025-10-10

**Authors:** Carlos Araujo, Maria Angelica Araujo, César Augusto Magalhães Benfatti, Anderson Camargo Moreira, Celso Peres Fernandes, Roberta Michels

**Affiliations:** 1 Department of Prosthodontics, Faculty of Dentistry (FOP), University of São Paulo (USP), Bauru 17012-901, Brazil; carlos@fob.usp.br; 2 Latin-America for Dentistry Education and Research (ILAPEO), Curitiba 80440-020, Brazil; angelicarehder@gmail.com; 3 Center for Education and Research on Dental Implants (CEPID), Post-Graduate Program in Dentistry (PPGO), Federal University of Santa Catarina (UFSC), Florianopolis 88040-900, Brazil; cesar.benfatti@ufsc.br; 4 LMPT Laboratory, Mechanical Engineering Department, Federal University of Santa Catarina (UFSC), Florianopolis 88040-900, Brazil; anderson@lmpt.ufsc.br (A.C.M.); celso@lmpt.ufsc.br (C.P.F.); 5 Department of Periodontology, School of Dental Medicine, University of Bern, 3010 Bern, Switzerland

**Keywords:** immediate dental implant loading, osseointegration, animal model, histology

## Abstract

**Background:** Dental implants are widely used to replace missing teeth, particularly in aesthetically sensitive areas. The implant’s macrogeometry is crucial for ensuring primary stability and successful osseointegration. Internal conical connections and reactive surfaces on implants have shown positive outcomes in tissue and bone stability. In response, a hybrid conical dental implant was designed to address a variety of clinical scenarios. **Materials and Methods:** This pilot study evaluated the performance of the hybrid conical implant using histological and micro-CT analysis in a preclinical model with immediate loading. Five implants were placed in a mongrel dog, and histomorphometric and micro-CT assessments were performed after 60 days of healing. **Results:** Analysis showed a high degree of osseointegration, with BIC at 61.56% and BT/TV at 77%. Micro-CT confirmed these findings, with nBIC at 82.20%. Vertical measurements indicated stable crestal bone. Peri-implant tissue displayed organized supracrestal connective tissue, without signs of inflammation or bone saucerization. Polarized light microscopy revealed collagen fibers in perpendicular and oblique orientations around the abutment, suggesting mechanical integration and biological sealing despite the absence of a prosthetic crown. **Conclusions:** Within the limitations of this exploratory study with one animal study, the hybrid conical implant showed favorable biological and structural responses under immediate loading. These preliminary findings provide useful insights for the refinement of implant design, although further investigations in larger preclinical and clinical studies are required before clinical applicability can be confirmed.

## 1. Introduction

Dental implants are a widely accepted and effective method for replacing lost teeth. For aesthetic zones, like the front teeth or areas prominently seen when smiling, careful consideration is necessary when selecting the right implant shape. In recent decades, numerous studies have highlighted the importance of dental implant design macrogeometry in securing primary stability for immediate loading and osseointegration [1,2,3,4,5,6,7]. In aesthetic regions, the coronal portions of implants can also influence soft tissue stability, including the emergence profile, papilla formation, and soft tissue adaptation [7].

The interface design between implants and abutments is a highly debated topic. Internal conical connections or tapered junctions show better results for tissue stability and mechanical performance [7,8,9,10]. Additionally, creating reactive implant surfaces has proven very effective in speeding up bone formation and enhancing bone-to-implant contact [11,12]. Research shows that surface roughness, which promotes pits and micro- or nano-cavitation, along with treatments that reduce blood surface tension by creating a specifically wet surface, can accelerate osseointegration [13].

Several studies have shown that the implant-abutment connection is crucial for peri-implant tissue stability. A systematic review and meta-analysis found that internal and conical connections are linked to less peri-implant bone loss compared to external ones, highlighting the importance of connection design in long-term results [14]. Likewise, experimental research has indicated that implant neck surface and placement depth can significantly impact crestal bone remodeling, especially when platform-switching designs are used [15]. These findings underscore that modifications at the implant-abutment interface are not only mechanical but also biological factors affecting osseointegration and marginal bone preservation.

Furthermore, histologic and animal studies have offered more insights into how abutment manipulation, connection geometry, and loading protocols influence peri-implant bone response. For example, experimental research in monkeys showed no significant differences between immediate and delayed loading of non-splinted implants, suggesting that peri-implant bone maintenance may rely more on interface design than on the loading protocol alone [16]. Recently, focus has shifted to abutment material and trans-mucosal configurations, which could predispose implants to peri-implantitis when suboptimal designs are used [17]. Canine models also indicate that repeated abutment disconnection and reconnection, along with surface modifications, can worsen crestal bone remodeling, emphasizing the need for innovations that reduce biological disruption at the implant-abutment junction [18]. Collectively, these findings support exploring new implant-abutment interface designs to enhance long-term peri-implant tissue stability.

Recent advances in implantology have led to significant progress in dental implants over the past few decades. As a result, a new type of dental implant, known today as the hybrid conical dental implant (Helix, Acqua^®^—Straumann/Neodent, Curitiba, Brazil), has been developed to address various clinical needs [19]. The implant is distinguished by its unique design, featuring a hybrid profile: cylindrical in the coronal third and conical in the more apical two-thirds. It also has sharp cutting threads at the apex that transition to square threads along its body, moving towards the crown. This design allows the implant to effectively cut and penetrate dense bone with its cutting edge while also compacting softer bone in the upper region. Consequently, it can be used across most bone densities with only minor adjustments to the drilling protocol.

This pilot study aims to demonstrate the performance of a new implant design using histological and micro-CT scan analyses in an animal model with immediate loading. Additionally, polarized light microscopy is employed to evaluate the properties of the surrounding soft tissue.

## 2. Materials and Methods

### 2.1. Ethical Aspects

The Ethics Committee on the Use of Animals (CEUA) at the Bauru School of Dentistry (FOB), University of São Paulo (USP) in Bauru, SP, Brazil, approved the animal experiments (No. 10/2009), which were carried out following the ARRIVE guidelines [15]. For this study, one mongrel dog was chosen. The use of a single animal was approved considering the pilot nature of the study and ethical guidelines to minimize animal use.

### 2.2. Animal

One mongrel dog (aged approximately 18 months and weighing 16 kg) was selected for this study. The dog had intact teeth and a healthy periodontal status. The dog was kept at a section of the Bauru School of Dentistry—USP animal facility (Bauru, São Paulo, Brazil) under lab conditions, with a temperature of 15 to 23 °C and humidity above 33%. The dog had unrestricted access to tap water and a laboratory diet.

### 2.3. Surgical Procedures

One hour before surgery, the dog received an intramuscular injection of penicillin and streptomycin (Pentabiótico; Fort Dodge-Pfizer, Campinas, São Paulo, Brazil) at 0.1 mL/kg, with the dose repeated five days after the surgery. For sedation, Acepromazine 0.2% (Acepran 0.2%; Univet, São Paulo, Brazil) was administered intramuscularly at 0.1–0.2 mg/kg to promote muscle relaxation and facilitate anesthesia. Additionally, Diazepam (Diempax; Sanofi-Aventis, Suzano, Brazil) was given intravenously at 0.25–0.50 mg/kg to assist with muscle relaxation and postoperative pain control. General anesthesia was induced with Propofol (Rapinovet; Schering-Plough, Millsboro, DE, USA) at 5 mg/kg, then maintained using 1–2% isoflurane in oxygen as needed. Postoperative care included administration of analgesics (buprenorphine and dipyrone, veterinary formulations) to control pain. A hygiene protocol with topical chlorhexidine and soft diet was followed during the healing period.

**Figure 1:**
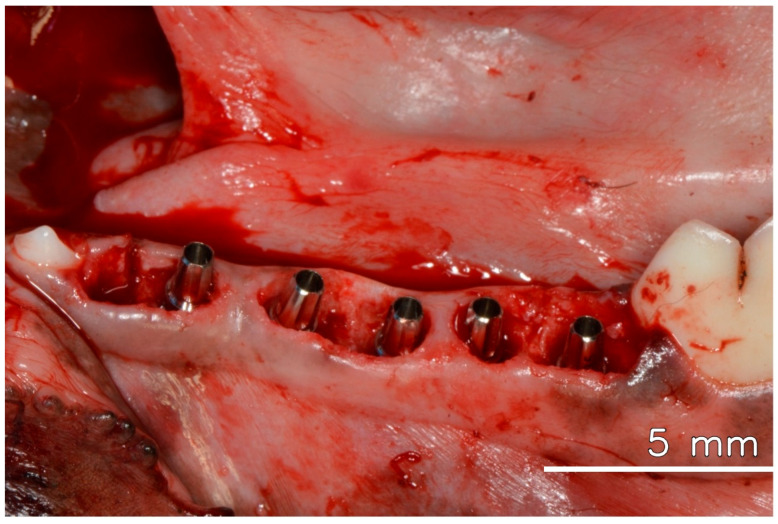
Dental implants are installed in the fresh extraction socket immediately after tooth extraction, with their respective abutments.

After anesthesia and preparation of the animal, three mandibular premolars were extracted. Five immediate implants, each with a diameter of 3.5 mm and a length of 10 mm, were placed in the extraction sockets and adjacent available areas, all meticulously positioned 2 mm below the bone level. All implants showed stability with an insertion torque of 45 N/cm or higher. Universal abutments (Straumann Neodent GM), with a prosthetic diameter of 3.3 mm, a transgingival height of 2.5 mm, and a prosthetic length of 6 mm, were promptly installed and torqued to 20 N/cm, following the manufacturer’s instructions, which allowed for immediate loading, even without the installation of crowns [2] (see Figure 1).

Euthanasia was performed 60 days (8 weeks) after implant insertion through an overdose of anesthetic, with tissue samples collected for histological and micro-CT analysis, fixed in 4% buffered formaldehyde.

### 2.4. Histological Process

All specimens were rinsed in saline and fixed in 4% buffered formaldehyde. They were then dehydrated through a graded alcohol series and embedded in resin (Technovit 7200 VLC + BPO; Kulzer & Co., Wehrheim, Germany). After polymerization, the blocks were processed with Exakt cutting and grinding tools (EXAKT, Apparatebau GmbH, Norderstedt, Germany). Thin sections, cut parallel to the sagittal plane (from buccal to distal aspect) passing through the implant center, were stained with Levai-Laczko dye. Sections approximately 100 μm thick were scanned using an Olympus BX61VS digital microscopy system with a 20× objective, resulting in a resolution of 0.32 μm per pixel. Histomorphometric analysis was conducted using Zeiss Efficient Navigation Pro software (ZEN 3.3 blue edition, Carl Zeiss Microscopy GmbH, Jena, Germany). Measurements of BIC were taken from the buccal and lingual sides of the implant and the apical region.

### 2.5. Micro-CT

Following the embedding process, all specimens underwent scanning via micro-computerized tomography (Micro-CT) using the high-resolution Zeiss/Xradia Versa system XRM-500 (Carl Zeiss X-ray Microscopy, Inc., Pleasanton, CA, USA). Image reconstruction was performed in both 2D and 3D. Image quantifications were executed using ImageJ/FIJI 15 (National Institutes of Health, Bethesda, MD, USA) and Avizo software version 2021.2 (Thermo Fisher Scientific, Waltham, MA, USA). Values were obtained for nBIC (the area between the implant and bone) and BT/TV (the bone volume fraction within 200 μm of the implant surface).

### 2.6. Histomorphometry

Histomorphometry analysis was conducted with Zeiss Efficient Navigation Pro software (Zen Pro, Carl Zeiss). All histomorphometric measurements for BIC were taken on both the buccal and lingual sides of the implant surface, focusing on the apical portion. Measurements were obtained in a vertical direction using two morphometric parameters: (1) the distance from the implant shoulder (IS) to the first BIC (IS-fBIC) and (2) the distance from the IS to the highest point of the bone crest (IS-BC). Negative values (-) were assigned when IS-fBIC or IS-BC was situated apical to the IS. Parameters for soft tissue measurements included: (1) the distance from the bone crest (BC) to the soft tissue margin (STM), (2) the distance from the abutment shoulder (AS) to the STM, and (3) the distance from fBIC to the TM.

### 2.7. Polarized Images

Polarized images were taken from non-stained samples using an Olympus^TM^ BX50 light microscope (Olympus Corporation, Tokyo, Japan). Photographs were captured at 4× and 40× magnifications (Olympus DP 71, Tokyo, Japan) with the image capture software, DP Controller 3.2.1.276 (2001–2006, Olympus Corporation, Tokyo, Japan), featuring image size specifications of 4080 × 3072 pixels and a spot size of 0.1%.

### 2.8. Statistical Analysis

Descriptive statistics were applied, and only mean values and standard deviations were calculated for all parameters in each sample using Microsoft Excel (Version [16.93.1]). No inferential statistical tests or power analysis were performed, as the study was limited to a single-animal pilot model with five implants. Consequently, the results should be interpreted with caution, acknowledging that the absence of a larger sample size and control group precludes robust statistical comparisons and limits the generalizability of the findings.

## 3. Results

**Figure 2:**
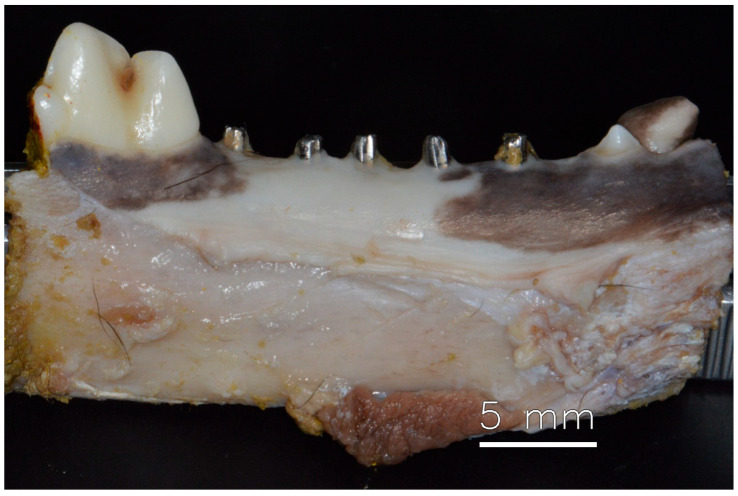
Mandible with healed soft tissue after 60 days of installation of dental implants.

After 60 days of healing, the dog was euthanized, and no problems could be observed in the appearance of the soft tissue healing. However, it was possible to identify damage to the abutments caused by mastication (Figure 2) demonstrating effective mastication load.

### 3.1. Histomorphometry and Micro-CT

Histomorphometric analysis revealed a mean BIC of 61.56% (SD = 4.06), indicating consistent osseointegration across samples. In the micro-CT analysis, the average nBIC was 82.20% with an SD of 73.55. For BT/TV, the mean was 77%, with a standard deviation of 9 (see Table 1).

Vertical measurements were conducted on both the lingual and buccal sides of each specimen, recording the distance from the first bone implant contact (fBIC) to the most coronal bone level (bone crest) relative to the implant shoulder (IS-fBIC/IS-BC) (see Table 1). Overall, the average was −0.69 mm on the buccal side and −0.33 mm on the lingual side for IS-fBIC. The mean IS-BC was 1.48 mm for the buccal aspect and 1.93 mm for the lingual aspect.

**Figure 3:**
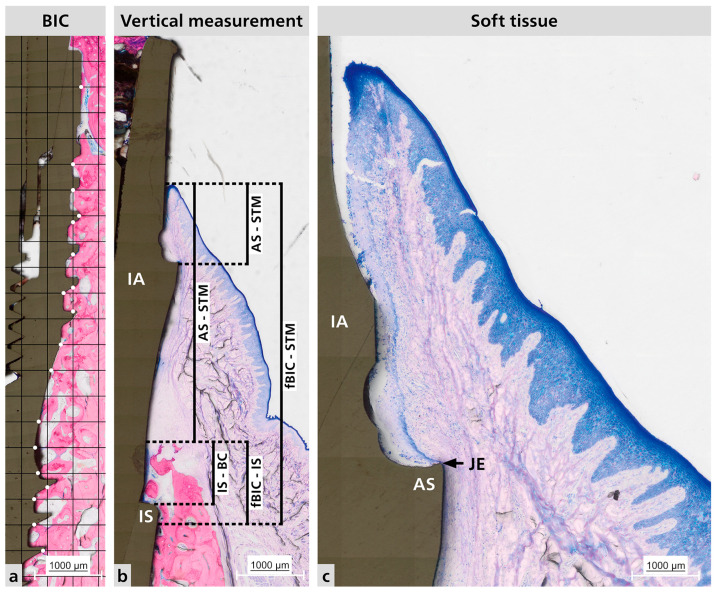
Representative histological sections. (**a**) Representative BIC measurement analysis. Where the white dots it is the contact between the bone and the implant surface. (**b**) Vertical measurements made for hard tissue and soft tissue; Distance between IS to fBIC and IS to BC; Distance between BC to STM, AS to STM, and fBIC to STM. (**c**) Peri-implant mucosa adaptation. BIC = bone to implant contact; IA = implant abutment; IS = implant shoulder; AS = abutment shoulder; STM = soft tissue margin; JE = junctional epithelium; fBIC = first bone to implant contact; BC = bone crest.

Although histomorphometric analysis could not measure the initial distance between the bone crest and the implant shoulder, all implants were installed with a manufacturer-provided key marker, confirming placement 2 mm below the bone crest. Histological examination showed no significant changes in this relationship that would indicate bone saucerization (Figure 3a).

Vertical measurements were taken on both the lingual and buccal sides of each specimen. The recorded parameter was the distance from the most coronal soft tissue margin (TM) to the bone crest (BC), abutment shoulder (AS), and fBIC (see Table 2).

### 3.2. Vertical Measurements

Histological analysis of the samples revealed no signs of peri-implant disease or infiltrated inflammatory cells. A mature connective soft tissue with a significant amount of collagen fibers was present around the abutment (Figure 3b,c). Additionally, the junctional epithelium (JE) attached to the abutment shoulder appears to be short.

**Figure 4:**
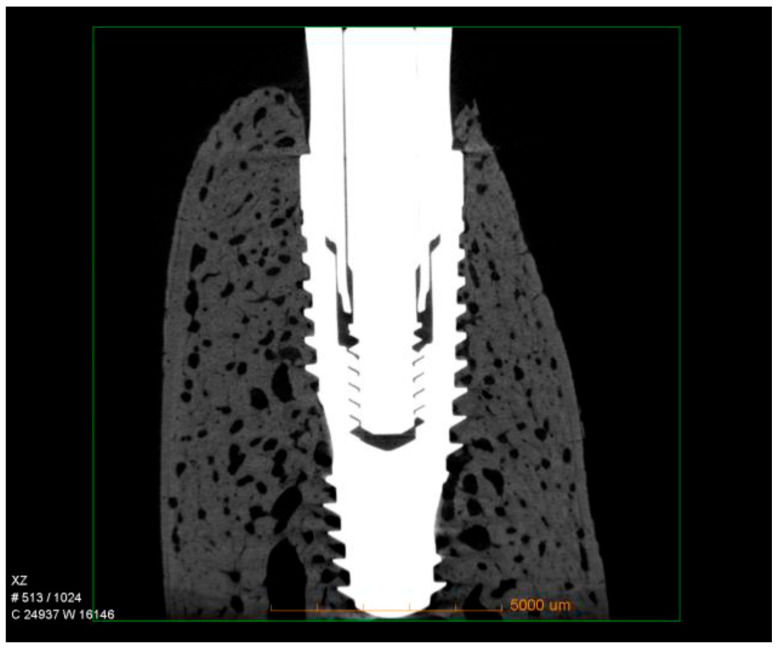
Representative micro-CT images of hybrid conical implants after 60 days of healing. The reconstructions show dense trabecular bone surrounding the implant surface, with preservation of crestal bone levels and no signs of marginal bone loss.

Micro-CT qualitative analysis confirmed close bone contact along the implant surface, with dense trabecular bone filling the peri-implant region. Three-dimensional reconstructions revealed that the hybrid conical implant maintained stable crestal bone levels, consistent with the histological findings. No evidence of bone resorption, marginal defects, or saucerization was observed. Instead, the peri-implant bone appeared continuous and well-integrated around the implant threads, particularly in the coronal portion, supporting the quantitative data obtained for nBIC and BT/TV (Figure 4).

### 3.3. Polarized Light

**Figure 5:**
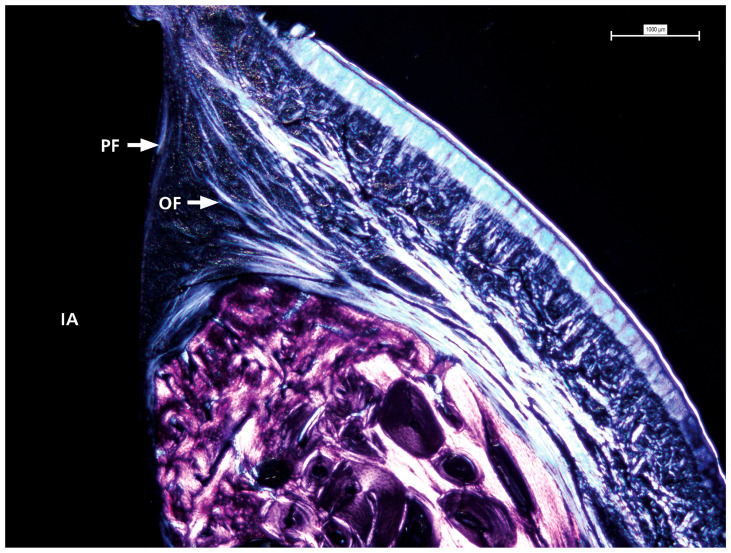
The detailed view shows the presence and distribution of collagen fibers with visible oblique and perpendicular arrangements in contact with the prosthetic abutment. IA = implant abutment; PF = perpendicular fibers; OF = oblique fibers.

Polarized light microscopy was used to examine the characteristics of the surrounding soft tissue (see Figure 5). This analysis consistently revealed a pattern across all samples in the distribution and orientation of collagen fibers around dental implant abutments. These fiber bundles were mainly associated with the abutment surface, with most samples showing complete bone coverage of the implant-abutment interface. The fibers originate from the nearby connective tissue, and their arrangement is more complex than a simple circular pattern. Instead, they are oriented obliquely and perpendicularly to the abutment surface, indicating a dynamic interaction between the abutment and soft tissue. This organization may influence the stability and adaptation of the soft tissue around the implant.

## 4. Discussion

This preclinical pilot study evaluated the performance of a hybrid implant design (Straumann/Neodent Helix GrandMorse^®^) under immediate placement and loading in a canine model. The aim was to assess peri-implant hard and soft tissue responses through histologic and micro-CT analysis. The findings provide early data contributing to the ongoing exploration of how macrogeometry, surface treatment, and connection design affect implant outcomes.

While there is now broad agreement on the clinical viability of various implant placement and loading protocols, including immediate approaches under certain case conditions [17,18], preclinical studies continue to be essential for testing new designs in controlled settings. The 2023 ITI Consensus Report [19] indicates that immediate placement and loading can be successful when high primary stability, correct implant placement, and effective soft tissue management are achieved. Therefore, our results reinforce existing evidence that a well-executed immediate protocol can produce positive biological outcomes, even in difficult circumstances.

Crestal bone levels remained stable 60 days post-surgery, with mean BC-IS distances of 1.48 mm buccally and 1.93 mm lingually, in line with the subcrestal 2 mm installation depth. This result is consistent with findings by Caricasulo et al. [9], who showed that internal conical connections contribute to bone stability by enhancing load distribution and sealing the implant-abutment interface. Furthermore, the maintenance of the marginal bone may also be attributed to the 2.5 mm transmucosal abutments used, as highlighted by Muñoz et al. [18].

The presence of organized supracrestal connective tissue (SCT) surrounding the abutments was confirmed histologically, supporting the concept that connective tissue acts as a biological barrier protecting the peri-implant region from apical migration of the epithelium and bacterial challenges [17,20]. Vertical measurements (e.g., BC-TM, AS-TM, fBIC-TM) demonstrated consistent SCT dimensions, with values ranging from 6 to 9 mm, suggesting a stable soft tissue environment enhanced by the abutment design and surgical technique.

Polarized light microscopy revealed collagen fiber bundles arranged obliquely and perpendicularly at the abutment-tissue interface. This orientation is biologically significant because collagen fibers aligned transversely or obliquely can better resist compressive and tensile forces, thereby enhancing the biomechanical resilience of the peri-implant mucosa and contributing to soft tissue stability. These findings are consistent with prior investigations showing that collagen fiber orientation plays a key role in establishing a functional peri-implant barrier and in distributing mechanical loads [21,22]. Experimental models have further shown that immediately loaded implants can promote a higher proportion of transversely oriented collagen fibers, associated with improved mineralization and load adaptation of peri-implant bone [23]. More recent studies also emphasize that collagen organization around transmucosal abutments supports biological sealing and may protect against epithelial down-growth and microbial penetration [24]. Collectively, the collagen orientation observed in this study suggests that the hybrid conical implant design may foster favorable conditions for both biological integration and mechanical stability of peri-implant soft tissues.

In terms of osseointegration, BIC and nBIC values of 61.56% and 82.20%, respectively, and a BT/TV ratio of 77% further confirm favorable bone responses. These outcomes are in accordance with previous studies evaluating hydrophilic surfaces, such as those by Sartoretto et al. [25] and Sánchez-Puetate et al. [26], which reported accelerated bone formation and improved tissue–implant integration under similar surface characteristics.

Our findingsare also in agreement with earlier preclinical studies on immediate loading protocols. Zubery et al. [27] reported in a canine model that modular transitional implants under immediate loading achieved substantial bone-to-implant contact. Romanos et al. [28], in *Macaca fascicularis*, similarly observed that immediate loading did not compromise peri-implant bone healing, with osseointegration levels comparable to unloaded implants. More recently, Stokholm et al. [29] found no significant differences between immediate and delayed occlusal loading of non-splinted implants in monkeys, supporting the biological feasibility of immediate function when primary stability is achieved. In this context, the present study adds novel evidence on soft tissue collagen organization, complementing histological and micro-CT outcomes and reinforcing that immediate loading, under controlled conditions, can lead to favorable peri-implant hard and soft tissue responses.

Nevertheless, several limitations should be recognized. This study depended on a single animal model, and the findings should be viewed with caution. The lack of a control group restricts comparison. Moreover, the absence of histomorphometric baseline measurements immediately after implantation may restrict conclusions about bone remodeling processes. These limitations emphasize the need for further studies with larger sample sizes, controlled designs, and extended observation periods.

## 5. Conclusions

Within the limitations of this exploratory preclinical study, the hybrid conical implant demonstrated stable crestal bone, organized connective tissue, and favorable bone-to-implant contact under immediate placement of the implant and abutment exposure. These results suggest that design features such as subcrestal positioning, internal conical connection, and a hydrophilic surface may contribute to early biological integration. However, the findings should be interpreted with caution, as further studies with larger sample sizes and diverse conditions are necessary to validate and expand these observations. Additional preclinical and clinical research will be required before definitive conclusions regarding clinical performance can be drawn.

## Figures and Tables

**Table 1:** BIC from the histomorphometry.

	BIC%	nBIC%	BT/TV%		IS-fBIC (mm)	IS-BC (mm)
Mean (SD)	61.56 (4.06)	82.20 (73.55)	77 (9)	Buccal	−0.69 (0.64)	1.48 (0.43)
				Lingual	−0.33 (0.26)	1.93 (0.66)

Note: Mean and SD for BIC from the histomorphometry in percentage with the 5 samples. Mean and SD for nBIC (area between the implant surface and bone from micro-CT scan) and BT/TV (bone volume fraction) percentage with the 5 samples. Mean and SD for the distance between IS (implant shoulder) to fBIC (first bone to implant contact) and IS to BC (bone crest).

**Table 2:** Mean and SD for vertical measurements.

		BC-TM (mm)	AS-STM (mm)	fBIC-STM (mm)
Mean (SD)	Buccal	6 (0)	3 (3)	7 (4)
	Lingual	6 (6)	3 (2.77)	9 (0.86)

Note: Mean and SD (standard deviation) for the distance between BC (bone crest) to STM (soft tissue margin), AS (abutment shoulder) to STM, and fBIC (fist bone to implant contact) to STM. The measurements were performed for all samples in the buccal and lingual aspects.

## Data Availability

No new data were created.
